# Sero-epidemiology of human immunodeficiency virus, hepatitis B virus and hepatitis C virus: a cross-sectional survey in a rural setting of the West region of Cameroon

**DOI:** 10.11604/pamj.2017.28.201.12717

**Published:** 2017-11-06

**Authors:** Jobert Richie Nansseu, Descartes Maxime Mbogning, Gwladys Chavely Monamele, Stive Fokam Tamoh, Hortense Kamga Gonsu, Charles Kouanfack, Yves Nathan Yanwou, Zacharie Sando

**Affiliations:** 1 Faculty of Medicine and Biomedical Sciences, University of Yaoundé I, Yaoundé, Cameroon; 2 Department of Disease, Epidemics and Pandemics Control, Ministry of Public Health, Yaoundé, Cameroon; 3 Department of Microbiology and Parasitology, University of Buea, Buea, Cameroon; 4 Garoua Regional Hospital, Garoua, Cameroon; 5 Laboratory of Bacteriology, Yaoundé University Teaching Hospital, Yaoundé, Cameroon; 6 HIV Day Care Unit, Yaoundé Central Hospital, Yaoundé, Cameroon; 7 Department of Pathology, Yaoundé Gynaeco-Obstetrics and Pediatric Hospital, Yaoundé, Cameroon

**Keywords:** HBV, HCV, HIV, seroprevalence, Cameroon

## Abstract

**Introduction:**

Human immunodeficiency Virus (HIV), hepatitis B virus (HBV), and hepatitis C virus (HCV) are the three most common chronic viral infections worldwide, specifically in sub-Saharan Africa (SSA). This study aimed to determine the sero-epidemiology of HIV, HBV and HCV infections in a rural setting of the West region of Cameroon, a SSA country.

**Methods:**

We conducted a cross-sectional study from August 2 to 5, 2014 in the three health districts of the Menoua Division, West region of Cameroon. Sixteen villages were randomly selected. Participants were currently living in the Division at the time of the survey, and enrolled after they had provided a signed consent form. HIV screening used the “determine test” followed by Hexagon HIV for positive cases to the first assay. HBV and HCV were detected using DIASpot HBsAg and DIASpot HCV-Ab, respectively.

**Results:**

On the whole, 612 subjects consented to take part in this study, of whom 71.1% were females. Mean age of the study population was 45.3 ± 17.9 years. The seroprevalences of HIV, HBV and HCV infections were 1.0% (6/582), 4.5% (20/443) and 6.3% (23/365), respectively. The 41-50 years age group was the most represented among HIV-positive subjects. HBV prevalence was higher in the 21-30 years age group (13.4%), followed by the 51-60 years age group (7.8%), with a significant difference of prevalences among age groups (p = 0.002). All HCV-positive cases were above 40 years of age with a higher prevalence in the > 70 years age group (33.3%) followed by the 61-70 years age group (14.5%); there was a significant difference between the age groups (p = 0.001).

**Conclusion:**

The seroprevalences of HIV, HBV and HCV infections in the Menoua Division of the West region of Cameroon were 1.0%, 4.5% and 6.3%, respectively. Preventive measures against these health threats need to be reinforced in this setting.

## Introduction

Human immunodeficiency virus (HIV), hepatitis B virus (HBV) and hepatitis C virus (HCV) continue to exact a heavy toll of illness and death all over the world. Indeed, estimates from the Joint United Nations Programme on HIV/AIDS reveal that 38.7 million people were living with HIV in 2015, among whom 2.1 million became newly infected and 1.1 million died from AIDS-related illnesses [1]. In the same line, an estimated 240 million people have chronic liver infections due to HBV and more than 686,000 people die every year due to complications of HBV, including cirrhosis and liver cancer [2, 3]. According to the World Health Organization (WHO), between 130 and 150 million people are chronically infected with HCV globally and approximately 700,000 people are estimated to die from hepatitis C-related liver diseases each year in the world [3, 4]. These pathogens share similar transmission routes including sexual, blood-blood contact and injecting drug usage [5]. Africa has been hit hardest by the HIV pandemic and has the second highest HBV and HCV prevalences, following Asia [1, 2, 4,6]. Nearly 6.5 to 19 million people were living with HIV in sub-Saharan Africa (SSA) with 410,000 to 960,000 new infections and 330,000 to 470,000 related deaths during 2015 [1], whereas HBV infection in this region is highly endemic (> 8%) [2, 7]. There is a variation in HCV prevalence across Africa. In fact, HCV prevalence reported from 33 SSA countries ranged between 0.1 and 13.8%, with a mean of 3% [8]. When it comes to Cameroon, a Central African country, the prevalence of HIV infection was estimated at 4.3% according to the 2011 Demographic and Health Survey, though this feature may vary from one area to another [9]. Previous data on HBV suggest that Cameroon is a region of high endemicity, with a seroprevalence approaching 10% [10-13]. Concerning HCV infection, the related prevalence is reported around 13.8% in the country [8]. Each and every nation, especially those of the sub-Saharan African region, should have as its priorities to better understand and characterize the epidemiology of viral infections and related risk factors [14]. However, there is dearth of data on the prevalence of HIV, HBV and HCV in specific areas of Cameroon, especially in remote zones of the country. Willing to fill this gap, the present study was thus undertaken, the aim of which was to determine the sero-epidemiology of HIV, HBV and HCV infections among populations living in a rural setting of the West region of Cameroon.

## Methods

**Ethical considerations**: This study was granted an ethical clearance by the Cameroon Bioethics Initiative Ethics Review and Consultancy Committee (CAMBIN ERCC No. 1021). Additionally, institutional authorizations were obtained from different administrative, traditional and health authorities of the Menoua Division. All procedures used in this survey were in accordance with the current revision of the Helsinki Declaration. All aspects and procedures of the study were fully presented to each potential participant; a written and signed informed-consent form was provided by each volunteering participant or his/her guardian (for those ages below 18 years old) before inclusion in the study. Anonymity of participants and confidentiality of results were scrupulously respected.

**Study design and setting**: We conducted a cross-sectional study from August 2 to 5, 2014 in the Menoua Division, West region of Cameroon, one of the 10 administrative regions of the country. The Menoua Division is one of the 8 Divisions of the West region, with an estimated population of 372,244 people according to the last general population census conducted in 2006 [15]. It comprises three health districts: Dschang, Penka Michel and Santchou. The study was held during a health campaign organised by the *"Association des Etudiants en Médecine originaires de l'Ouest (AEMO)"*. Sixteen villages of the Division were randomly selected and visited during the campaign. Populations were previously informed of the day the medical team would visit their village for free medical consultations; those willing to consult and/or undergo free screenings (hypertension, diabetes, cervical cancer, HIV, HBV and HCV infections) could attend the health campaign. One health facility in the village offering sufficient space and adequate equipment was retained as the health campaign site in that specific village.

**Study participants**: We included persons residing in the Division since at least 6 months, who visited the medical team during the health campaign and who volunteered (or their guardians, for those aged less than 18 years) to take part in the study, irrespective of their age, sex, occupation or risk of exposure to HIV, HBV or HCV infections. All participants (or guardians) provided a written and signed informed consent form before inclusion in the study.

**Data collection and blood sampling**: Data were collected using a standardized and pre-tested questionnaire. After the medical consultation and pre-test counselling, participants accepting to be tested for at least one of the three pathogens were received at the laboratory and their socio-demographic characteristics (mainly age and sex) were recorded. Subsequently, blood was aseptically collected in a 4 ml EDTA tube; plasma was obtained after 30 minutes of natural sedimentation and used to screen for the different viral infections. Participants were free to undergo one or several laboratory exams. After the post-test counselling, participants with positive or discordant results were referred to specialised centres of the Division for further investigations and/or proper management. Participants with negative results were encouraged to continue and reinforce preventive methods.

**Screening of HIV, HBV and HCV**: HIV screening was performed using a highly sensitive assay: Determine HIV-1/2 (Abbott Laboratories, IL, USA), an immuno-chromatographic assay with a specificity of approximately 99.6% and a sensitivity approaching 99.4%. When this first test was positive, the Hexagon HIV (Human Diagnostics, Germany) was then used for confirmation: this is a rapid test based on an immuno-chromatographic technique, with a sensitivity of 100% and a specificity approaching 99.9%. All participants whose results were positive by both techniques were considered true positives; those negative to the Determine test were considered true negatives. Subjects presenting discordant results were excluded from further analyses; they were referred to a reference centre for confirmation and further management. HBV was detected using DIASpot HBsAg (DIASpot Diagnostics, USA) following the manufacturer's instructions. DIASpot HBsAg is a one-step immunoassay-based test for qualitative detection of hepatitis B surface antigen (HBsAg) in serum. This test has a relative sensitivity and specificity of 99% and 97.0%, respectively. IgG antibodies to HCV were detected using DIASpot HCV-Ab test strips (DIASpot Diagnostics, USA), an immuno-chromatographic assay that has a sensitivity of almost 99.9% and a specificity of 98.6%.

**Statistical methods**: Data were coded, entered and analysed using SPSS version 18.0 (SPSS, Inc, Chicago, Illinois, USA). Results are presented as count (proportion) for qualitative variables and mean ± standard deviation (SD) for quantitative ones. Differences in proportions among the different subgroups and other categorical variables were compared using the Chi-square test or Fisher's exact test, where appropriate. p values less than 0.05 were considered of statistical significance.

**Ethics approval and consent to participate**: An ethical clearance was issued by the Cameroon Bioethics Initiative Ethics Review and Consultancy Committee (CAMBIN ERCC No. 1021). Additionally, institutional authorizations were obtained from different administrative, traditional and health authorities of the Menoua Division. All aspects and procedures of the study were fully presented to each potential participant; a written and signed informed-consent form was provided by each volunteering participant or his/her guardian (for those ages below 18 years old) before inclusion in the study.

## Results

Overall, 612 subjects consented to take part in this study. The study population's distribution according to health districts was as follows: 419 (68.5%) from Dschang, 123 (20.1%) from Penka Michel and 70 (11.4%) from Santchou. There were 177 (28.9%) males, hence a male/female sex ratio of 0.4/1. Ages ranged from 1 to 92 years with a mean of 45.3 ± 17.9 years. Among the study population, 582 subjects were screened for HIV, 443 for HBV and 365 for HCV (Table 1). The seroprevalences of HIV, HBV and HCV were 1.0% (6/582; 95% confidence interval (CI): 0.2-1.9%), 4.5% (20/443; 95% CI: 2.6-6.4%) and 6.3% (23/365; 95% CI: 3.8-8.8%), respectively. The 41-50 years age group was the most represented among HIV-positive subjects (Table 2). HBV prevalence was higher in the 21-30 years age group (13.4%), followed by the 51-60 years age group (7.8%). The difference in HBV prevalence within the age groups was statistically significant (p = 0.002; Table 2). All HCV-positive cases were above 40 years of age with a higher prevalence in the > 70 years age group (33.3%), followed by the 61-70 years age group (14.5%). HCV prevalence was significantly different with respect to age groups (p = 0.001; Table 2). On the contrary, there was no difference in the presence of each of these three pathogens with regard to sex (Table 2). There was one case of HBV/HCV co-infection; neither case of co-infection with HIV nor triple infection was recorded.

**Table 1:** Age distribution of the study population

Age group	(n/612)	%	HIV	HBV	HCV
< 20	58	9.48	54	45	36
21-30	87	14.21	83	67	56
31-40	100	16.34	95	68	61
41-50	118	19.28	111	82	63
51-60	109	17.81	104	77	62
61-70	100	16.34	96	79	69
> 70	40	6.54	39	25	18
Total	612		582	443	365

**Table 2:** Prevalence of HIV, HBV and HCV based on socio-demographic data

Variable	HIV+ N (%)	*p* value	HBV+ N (%)	*p* value	HCV+ N (%)	*p* value
**Age**						
< 20	0	0.539	1 (2.2)	0.002	0	0.001
21-30	1 (1.2)		9 (13.4)		0	
31-40	1 (1.1)		0		0	
41-50	3 (2.7)		3 (3.7)		3 (4.8)	
51-60	1 (1.0)		6 (7.8)		4 (6.5)	
61-70	0		1 (1.3)		10 (14.5)	
> 70	0		0		6 (33.3)	
**Sex**						
Male	2 (1.2)	0.559	7 (5.2)	0.606	7 (6.7)	0.798
Female	4 (1.0)		13 (4.1)		16 (6.0)	

## Discussion

For a proper control of blood borne infections, it is essential for appropriate measures to be taken not only in the urban settings but also in rural areas. Traditional beliefs and lack of communication facilities impede the surveillance of these infections within these latter regions. Moreover, in rural areas, with limited access to health facilities and educational programs, blood borne-viruses are more prone to infect individuals [16]. Previous reports from the 2011 Cameroon Demographic and Health Survey estimated HIV prevalence in the Western region at 2.8% on one hand and 3.8% in rural settings on the other hand, which are both lower than the 4.3% prevalence in the national population [9]. A similar scenario was reported from a rural setting in Nigeria where a lower prevalence of 2.4% was observed when compared to the general population (9.7%) [17] and comparable to the 2.5% prevalence observed by Noubiap et al among a rural subset of pregnant women [18]. Mirroring our 1.0%, a prevalence of 1.1% was observed in 2014 among university students in the Western region of Cameroon [19]. The low prevalence of HIV infection can be explained by the prevention programmes that have been instituted in the country during this last decade. Evidence from the literature has brought to consider Cameroon as a region of high endemicity with regard to HBV infection. Indeed, Chiaramonte et al reported a 19.9% prevalence among school children in an urban setting in 1991 [10], while Foupouapouognigni et al, Brennan et al and Noubiap et al found respectively 11.8%, 10.5% and 10.1%, in adult populations [11-13]. However, our HBV infection prevalence was lower (4.5%). In accordance with these findings, Sobze et al reported a 2.8% prevalence in the West region of Cameroon in 2014 [19]. The majority of positive HBV cases were from the 21-30 years age group. This points out that HBV was probably acquired by this population by sexual route or during childhood, as these are predictors of chronic HBV infection in endemic regions [20, 21]. The Global Advisory Group on the Expanded Programme on Immunization recommended that countries with a more than 2% prevalence of HBV carriers should add hepatitis B vaccine into their routine infant immunization schedules [22]. With the implementation of hepattis B vaccine in the Expanded Programme of Immunzation in Cameroon in 2005, a decrease in the trends of hepatitis B is expected in future decades. Education campaigns and vaccination of the unimmunized population would be worthcoming to curb HBV-transmission and reduce its prevalence.

A high HCV prevalence of 6.3% was observed within our study population. Noubiap et al in Edea reported a similarly high prevalence of 4.8% among blood donors [13]. Higher seroprevalence rates have been reported in other studies: 12.4% in Nigeria [17], 14.7% in Egypt [23], 17.1% in Cameroon [24] and 20.7% in Gabon [25]. These results are contradictory to reports from other studies in Africa where lower prevalence values between 0.6% and 1.2% were reported [11, 14, 26]. Age specific HCV prevalence was observed in this study. All individuals positive for HCV infection were above 40 years of age with an increase in seroprevalence from 6.3% to 10.8% after age 40. This is similar to previous findings in other rural regions of Cameroon [24, 27, 28], Gabon [25] and Libya [14]. Medical interventions (such as intravenous antimalarial drugs, transfusions) and traditional practices (circumcision) can be hypothesized for the high prevalence of HCV among the older age group as reported by Pépin et al [29]. This enforces the recommendation from the Centre for Disease Control and Prevention, stating that everyone born during 1945 to 1965 should be tested at least once for HCV [5]. Further investigations need to be carried out in order to determine the risk factors associated with the high HCV prevalence within this population. There was only one case of HBV/HCV co-infection which is controversial to reports from Barth et al. who figured out high cases of HIV co-infections with HBV or HCV [30]. This was also observed by Noubiap et al [13]. This may be due to the relative low prevalence of HIV and HBV encountered in this study. Unfortunately, this study has some limitations, especially the accuracy of the assays used in screening for HBV and HCV. Indeed, rapid tests were used which have a relatively poor sensitivity as compared to Enzyme immunoassays (EIA) for HBsAg and anti-HCV screening. Due to the lack of a confirmatory test for HBV and HCV screening, the prevalences obtained might have been consequently overestimated through inclusion of some false positive cases. Another limitation of this study lies in the fact that our participants were not randomly selected among the entire population of the Menoua Division, though the villages included were randomly chosen. Therefore, our results may not be a true reflect of the real burden of these threats in the Menoua Division. Additionally, the cross-sectional design of this study impeded to seek for risk factors associated to HIV, HBV and HCV infections in this population. Further studies are warranted in this regard.

## Conclusion

Results from the present study show that, the sero-prevalences of HIV, HBV and HCV infections in the Menoua Division of the West Region of Cameroon were respectively, 1.0%, 4.5% and 6.3%. Although we observed a significant difference of prevalences among age groups in case of HBV and HCV infections, future studies are warranted to fully investigate the various risk factors that impact the occurrence of these viral infections in this setting. Preventive measures towards these threats need to be reinforced in this population: unimmunized individuals should be vaccinated against HBV; adults aged 40 years and above should be screened for HCV infection and closely followed-up if found infected; and educational sessions advocating the ABC strategy should be reinforced with regard to HIV infection.

### What is known about this topic

- Human immunodeficiency Virus (HIV), hepatitis B virus (HBV) and hepatitis C virus (HCV) are the most common chronic viral infections worldwide;
- Sub-Saharan Africa including Cameroon carries the heaviest burden of these viral infections.

### What this study adds

- The sero-prevalences of HIV, HBV and HCV infections in the Menoua Division of the West Region of Cameroon were respectively, 1.0%, 4.5% and 6.3%;
- People aged 21-30 years were significantly more infected with HBV than their counterparts;
- HCV infection was significantly more prevalent in those aged >70 years and 61-70 years.

## Competing interests

The authors declare no competing interests.
